# Quality of Acute Stroke Care at Primary Stroke Centers Before and After Certification in Comparison to Never-Certified Hospitals

**DOI:** 10.3389/fneur.2019.01396

**Published:** 2020-01-22

**Authors:** Kristina Shkirkova, Theodore T. Wang, Lily Vartanyan, David S. Liebeskind, Marc Eckstein, Sidney Starkman, Samuel Stratton, Franklin D. Pratt, Scott Hamilton, May Kim-Tenser, Robin Conwit, Jeffrey L. Saver, Nerses Sanossian

**Affiliations:** ^1^Zilkha Neurogenetic Institute, Keck School of Medicine, University of Southern California, Los Angeles, CA, United States; ^2^Department of Neurology, Roxanna Todd Hodges Comprehensive Stroke Center, Keck School of Medicine, University of Southern California, Los Angeles, CA, United States; ^3^Department of Neurology, UCLA Comprehensive Stroke Center, Los Angeles, CA, United States; ^4^Department of Emergency Medicine, University of Southern California, Los Angeles, CA, United States; ^5^Department of Community Health Sciences, University of California, Los Angeles, Los Angeles, CA, United States; ^6^Los Angeles County Department of Public Health, Los Angeles, CA, United States; ^7^Department of Neurology, Stanford Stroke Center, School of Medicine, Stanford University, Palo Alto, CA, United States; ^8^National Institutes of Health, Bethesda, MD, United States

**Keywords:** stroke, primary stroke center, stroke center certification, acute ischemic stroke, cerebral ischemia, tPA, thrombolytic

## Abstract

**Background and Purpose:** Primary stroke center (PSC) certification is associated with improvements in stroke care and outcome. However, these improvements may reflect a higher baseline level of care delivery in hospitals eventually achieving certification. This study examines whether advancements in acute stroke care at PSCs are due to certification or factors intrinsic to the hospital.

**Methods:** Data was obtained from the Field Administration of Stroke Therapy-Magnesium (FAST-MAG) trial with participation of 40 Emergency Medical System agencies, 315 ambulances, and 60 acute receiving hospitals in Los Angeles and Orange Counties. Subjects were transported to one of three types of destinations: PSC certified hospitals (PSCs), hospitals that were not PSCs at time of enrollment but would later become certified (pre-PSCs), and hospitals that would never be certified (non-PSCs). Metrics of acute stroke care quality included time arrival to imaging, use of intravenous tPA, and arrival to treatment.

**Results:** Of 1,700 cases, 856(50%) were at certified PSCs, 529(31%) were at pre-PSCs, and 315 (19%) were at non-PSCs. Mean (SD) was 33min (±76.1) at PSCs, 47(±86.6) at pre-PSCs, and 49(±71.7) at non-PSCs. Of 1,223 cerebral ischemia cases, rate of tPA utilization was 43% at PSCs, 27% at pre-PSCs, and 28% at non-PSCs. Mean ED arrival to thrombolysis was 71(±32.7) at PSC, 98(±37.6) at pre-PSC, and 95(±45.0) at non-PSCs. PSCs had improved time to imaging (*p* = 0.014), percent tPA use (*p* < 0.001), and time to treatment (*p* = 0.003).

**Conclusions:** Stroke care at hospitals prior to PSC certification is equivalent to care at non-PSCs.

**Clinical Trial Registration**: http://www.clinicaltrials.gov. Unique identifier: NCT00059332.

## Introduction

The Brain Attack Coalition (BAC) published their recommendations for the formation and operation of Primary Stroke Centers (PSCs) in 2000 and updated their recommendations in 2011 (1, 2). Multiple studies have demonstrated that PSC-certification is associated with improvement in stroke care, such as streamlined processes, improved rates of thrombolysis, and better outcomes (3–7). However, nationwide, less than one in three hospitals has achieved this designation (8). There are many barriers to becoming a PSC, including costs, lack of diversion of patients to appropriate levels of care, presence of leadership, and shortage of neurological expertise (1, 2, 9). Thus, many hospitals may never be able to achieve PSC certification.

There are challenges in demonstrating that benefits associated with PSCs are directly related to the certification process. Investigating outcomes at PSCs by doing pre- and post-certification comparisons is difficult for several reasons. Because the BAC criteria are organized around 11 aspects of stroke care, hospitals that are not fully certified can adopt many, but not all, elements of PSCs shown to be beneficial and improve overall stroke care (1, 2, 9). Additionally, improved outcomes at hospitals that achieve PSC certification may be explained by a higher baseline level of stroke care and expertise. We aimed to determine whether improvements in stroke care seen in PSCs in some part reflect the fact that hospitals eventually achieving certification are better at delivering care at baseline.

## Methods

Data was obtained from the National Institute of Health-NINDS-sponsored Field Administration of Stroke Therapy Magnesium (FAST-MAG) clinical trial conducted in Los Angeles County from 2005 to 2012 and in Orange County from 2010 to 2012 (10). FAST-MAG was a phase 3, randomized, placebo-controlled clinical trial of prehospital, field-initiated magnesium sulfate in hyperacute stroke patients presenting within the first 2 h of defined symptom 5 onset (11, 12). The study protocol was approved by the institutional review board at each prehospital and hospital study site.

The current study only included FAST-MAG trial patients from Los Angeles County, chosen because of its diverse population of inhabitants. Participating sites included 32 Emergency Medical System agencies, 228 paramedic ambulances, and 55 acute receiving hospitals in the County of Los Angeles. The 55 FAST-MAG consortium of acute receiving hospitals included community hospitals, regional centers, and tertiary academic centers. Patient enrollment criteria required that subjects be identified as likely having acute stroke by the Los Angeles Prehospital Stroke Screen (LAPSS) (13, 14), in addition to having a paramedic- and physician-confirmed last known well time (LKWT) within 2 h of actual last known well time.

During the FAST-MAG trial, many hospitals began the PSC certification process and eventually achieved status as PSC. Thus, each patient involved as a subject in the FAST-MAG trial was coded as enrolled at either a hospital already certified as a PSC by The Joint Commission (TJC) or an equivalent national body (8); a hospital that was not yet a PSC but would later become certified; or a hospital that never achieved certification. For the current study, subjects were classified as being enrolled at either a designated PSC (PSC), a hospital that was not a PSC at the time of enrollment but would later become certified (pre-PSC), or a hospital that would never achieve certification (non-PSC).

Three metrics were chosen to reflect quality of acute stroke care: time from Emergency Department (ED) arrival to first brain scan (“door to imaging”), rate of tPA utilization in cases of cerebral ischemia, and time from ED arrival to initiation of tPA (“door to tPA initiation”).

## Results

From January 2005 to December 2012, a total of 1,700 patients met criteria and were enrolled in the current study. 856 (50%) were coded as enrolled at certified PSCs, 529 (31%) were coded as enrolled at pre-PSCs, and 315 (19%) were coded as enrolled at non-PSCs (Table 1). Clinical characteristics of the overall FAST-MAG Study Population and PSC, pre-PSC, and non-PSC populations are listed in Table 2.

**Table 1:** Patient enrollment at PSCs, pre-PSCs, and non-PSCs.

**Characteristic**	**Enrolled, *n* (%)**
Certified PSC	856 (50)
Pre-PSC	529 (31)
Non-PSC	315 (19)
Total	1,700 (100)

**Table 2:** Patient characteristics of the overall FAST-MAG Study Population and PSC, pre-PSC, and non-PSC populations.

**Characteristic**	**All patients (*n* = 1,700)**	**PSC (*n* = 856)**	**pre-PSC (*n* = 529)**	**non-PSC (*n* = 315)**	***P*-value (between PSC, pre-PSC, non-PSC)**
Age, mean, in years (SD)	60.5 (14)	69.4 (3.6)	70.0 (12.9)	68.6 (14.2)	0.37
Females, *n* (%)	691 (42%)	375 (43.6)	226 (43.1)	124 (39.4)	0.42
Race					
White	1260 (78%)	660 (76.7)	437 (83.4)	228 (72.4)	<0.0001
Black	218 (13%)	117 (13.6)	40 (7.6)	62 (19.7)	
Asian	131 (8%)	75 (8.7)	41 (7.8)	23 (7.3)	
Other	18 (1%)	9 (1.0)	6 (1.2)	2 (0.6)	
Hispanic ethnicity	391 (24%)	203 (23.6)	109 (20.8)	90 (28.6)	0.037
Prehospital LAMS Score, Median (IQR)	4 (3–5)	4 (3–5)	4 (3–5)	4 (3–5)	0.73
Final diagnosis					
Cerebral ischemia	1191 (73%)	634 (73.6)	388 (74.2)	223 (70.8)	0.78
ICH	372 (23%)	191 (22.2)	116 (22.2)	80 (25.4)	
Stroke mimics	64 (4%)	36 (4.2)	19 (3.6)	12 (3.8)	
Time from 911 call to paramedic on-scene, minMedian (IQR)	6 (5–8)	6 (5–8)^*^	6 (5–8)**	6 (5–8)^*^/**	0.001
Time on-scene to ED arrival, minMedian (IQR)	33 (27–39)	32 (26–38)^*^	33 (28–39)**	34 (28–41)^*^/**	<0.0001
Intravenous tPA Administered (% of cerebral ischemia, N treated/N cerebral ischemia)	34% (408/1191)	44% (280/634)	27% (107/388)	28% (62/223)	<0.0001

LAMS: Los Angeles Motor Score (15); LKWT: Last known well time; tPA: Tissue plasminogen activator;

* *Significant Difference*.

**Figure 1:**
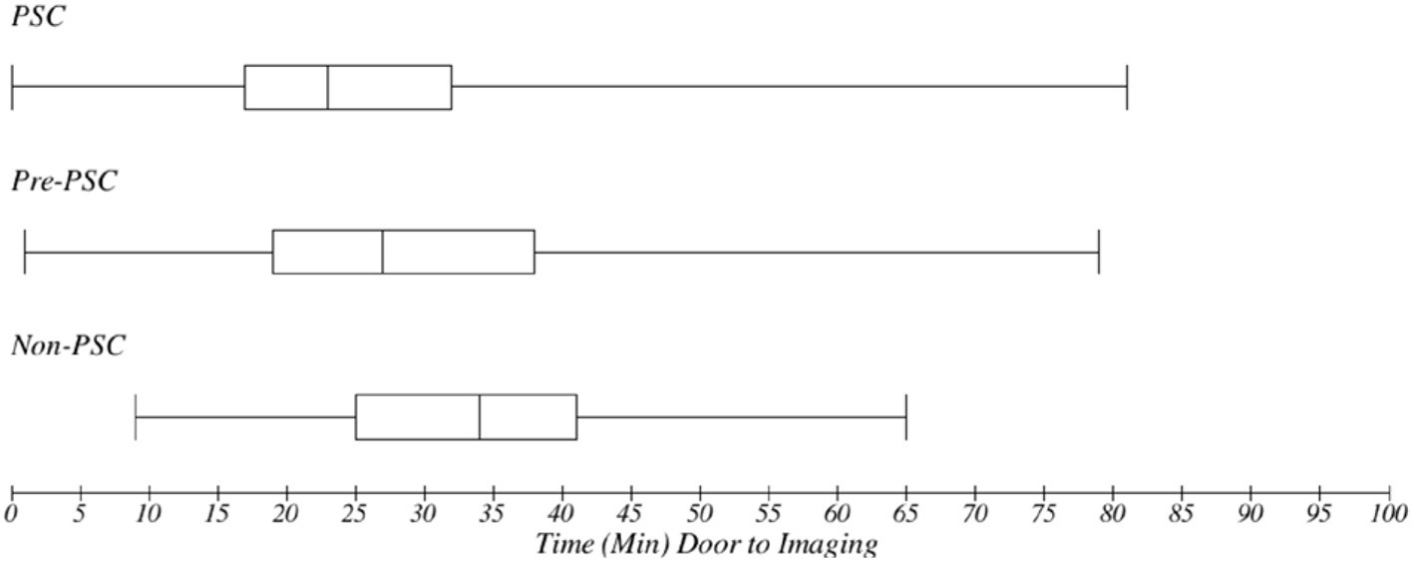
Metrics of acute stroke care quality: time (minutes) from door to imaging: median time with interquartile range in minutes from emergency department (ED) arrival (door) to first brain scan (imaging) was 23 (17–32) at PSCs, 27 (19–38) at pre-PSCs, and 34 (25–41) at non-PSCs (*p* < 0.001). PSCs had a faster time from ED arrival to first brain scan.

**Figure 2:**
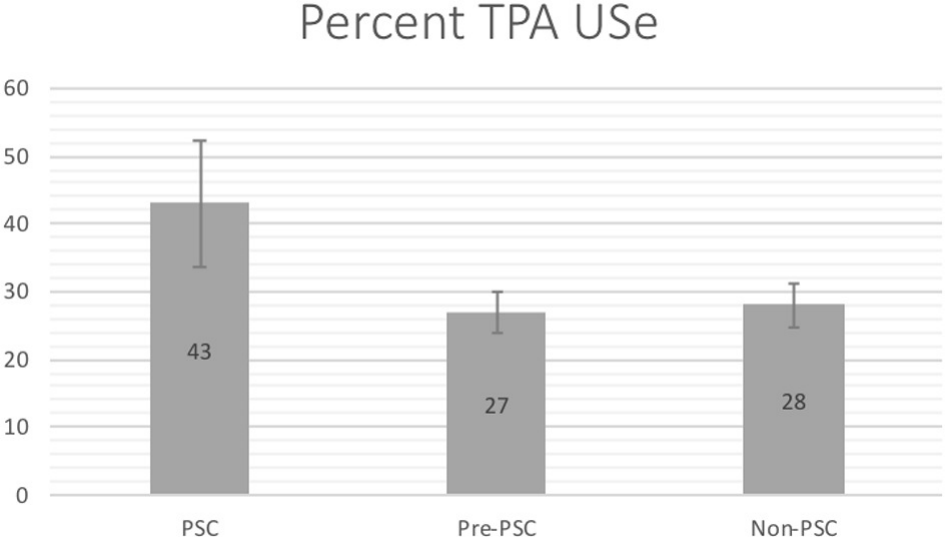
Metrics of acute stroke care quality: tPA use in cerebral ischemia: mean rates of tPA utilization in cases of cerebral ischemia. Of 1,223 cerebral ischemia cases, rate of tPA utilization was 43% at PSCs, 27% at pre-PSCs, and 28% at non-PSCs (*p* < 0.001, X^2^). PSCs had a higher rate of tPA utilization in patients diagnosed with ischemic stroke.

**Figure 3:**
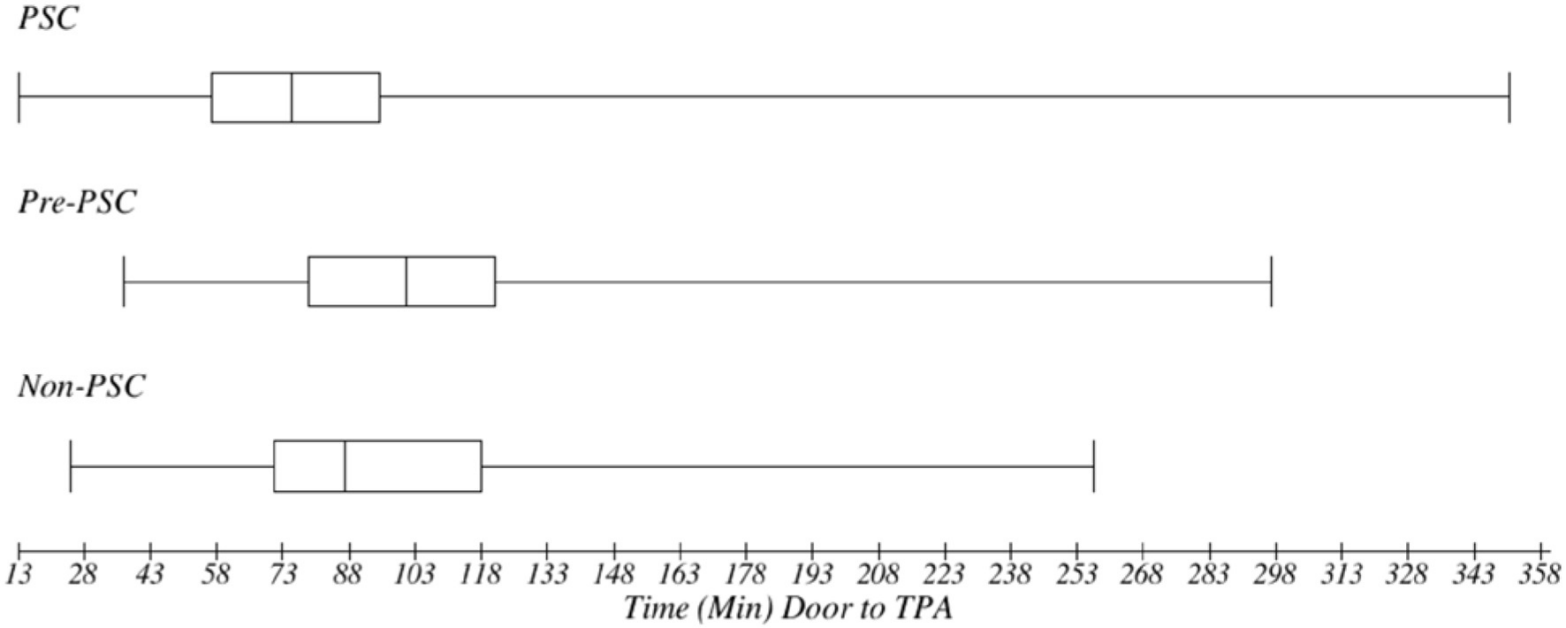
Metrics of acute stroke care quality: median time with interquartile range (minutes) from door to tPA initiation: in treated cerebral ischemia cases, time in minutes from ED arrival (door) to initiation of tPA was 75 (57–95) at PSC, 101 (79–121) at pre-PSC, and 87 (71–118) at non-PSCs (*p* < 0.001). PSCs had a reduced time from door to thrombolysis.

Median (IQR) time from arrival to imaging was 23 min (16–32) at PSCs, 33.5 (24–46) at pre-PSCs, and 37 (26–53) at non-PSCs (*p* < 0.0001) (Figure 1). Among cases of cerebral ischemia (*n* = 1,223), rates of tPA utilization were 43% at PSCs, 27% at pre-PSCs, and 28% at non-PSCs (*p* < 0.001, X^2^) (Figure 2). In treated cerebral ischemia cases, median (IQR) time in minutes from ED arrival (door) to initiation of tPA was 75 (57–95) at PSCs, 101 (79–121) at pre-PSCs, and 87 (71–118) at non-PSCs (*p* < 0.0001) (Figure 3).

As compared to hospitals that were not certified PSCs at the time of study enrollment but eventually achieved certification, hospitals that had already achieved PSC designation at the time of enrollment had a statistically significant reduction in the time from ED arrival to first brain scan, from 47 to 33 min (*p* = 0.014); a statistically significant increased rate of tPA utilization in cases of cerebral ischemia, from 27 to 43% (*p* < 0.001); and a statistically significant reduction in the time from ED arrival to tPA initiation, from 98 to 71 min (*p* = 0.003). In contrast, comparing values between non-PSCs (hospitals that would never achieved PSC status) and pre-PSCs (hospitals that eventually completed certification but were not PSCs at time of enrollment) revealed no significant difference in time to imaging (47 vs. 49 min), percent tPA use (27% vs. 28%), and time to thrombolysis (98 vs. 95 min).

## Discussion

Our findings in this study demonstrate that hospitals with PSC designation have shorter time of door to first imaging. Furthermore, in cerebral ischemia PSC hospitals had higher rate of tPA use in patients who met criteria for thrombolytic treatment as well as shorter time from door to needle. In contrast, the data reflect that stroke care at hospitals prior to PSC certification was equivalent to care at non-PSCs, hospitals that would never achieve certification. These findings suggest that improvements in acute stroke evaluation and care at PSCs are a result of undergoing the certification process and achieving designation, and not intrinsic to hospitals.

Prior studies attempting to evaluate this question have demonstrated mixed results. A 2009 study found that hospitals certified as PSCs had better patient outcomes as compared to non-certified hospitals at least 11 months before the Joint Commission certification program began (16). Outcomes studied included in-hospital mortality, 30-day mortality, and 30-day readmission, all of which were found to be lower in the future PSC-certified hospitals. This study was unable to take into account stroke severity potentially affecting outcomes, and they were unable to address thrombolytic use in stroke. The study coordinators concluded that cross-sectional analyses examining the benefits of stroke center certification have the potential to be biased if they do not account for these pre-existing differences.

A 2011 follow-up study by the same group again compared hospitals with and without PSC certification but analyzed 30-day risk-standardized mortality (RSMR) and readmission (RSRR) rates (17). Their results showed that ~50% of PSCs had lower than the national average mortality rate, vs. 19% of non-PSCs. However, their data on readmission rates did not demonstrate definitive improvement in PSCs. They concluded that while there were more high-performing hospitals identified in the PSC group, certification did not necessarily guarantee better performance at these sites.

In contrast, a 2011 New York State observational study evaluated if care at stroke centers had an effect on mortality, and found greater use of thrombolytic therapy and reduced mortality rates in patients admitted to PSCs when compared to patients admitted to non-designated hospitals (5). Notably, they were able to demonstrate that these outcome differences were specific to a diagnosis of stroke, vs. acute myocardial infarction and/or gastrointestinal hemorrhage. These findings suggest that improvements in stroke care are specific to PSC designation and the implementation of BAC stroke guidelines, rather than to factors intrinsic to hospitals achieving certification that affect overall quality improvement.

Although prior studies have shown that PSCs are associated with reduced mortality, increased use of thrombolytic therapy, and related processes of care and outcomes in acute stroke cases (6, 7), the results from these studies have failed to definitively demonstrate that improvements in stroke care are a result of the PSC certification process. This is the first study that we are aware of that examined hospitals at various stages of the certification process. By doing so, we were uniquely able to analyze data from pre-PSCs and non-PSCs to address the quality of stroke care at hospitals prior to PSC certification and compare it to the quality of stroke care at hospitals that would never achieve certification. Thus, by showing that care at pre-PSCs is equivalent to care at non-PSCs, the findings in our study support prior reports that the improvements seen in PSCs are derived directly from achieving designation.

Potential limitations exist in the present study. Data on acute stroke care was obtained from the FAST-MAG trial, which did not enroll patients transported to smaller hospitals, representing ~20% of the hospitals in Los Angeles County. Therefore, the findings in this study do not account for these patients. However, as the study progressed, and more hospitals became Approved Stroke Centers and joined the regional system, additional geographic areas were incorporated into the trial, covering a larger subset of patients in the county. None of the FAST-MAG trial exclusion criteria, such as renal failure, absence of a consent provider, or patient declination regarding trial participation, appear likely to confound analysis. Additionally, our study was limited to 3 metrics chosen to reflect acute stroke care and did not assess outcomes like stroke morbidity, mortality, and readmission rates. Future studies are needed to definitively address PSC certification on these patient outcomes. Finally, the BAC guidelines, our study, and most of the prior studies found in the literature focus primarily on acute ischemic stroke and initiation of thrombolytic therapy. Therefore, there is a deficit in research assessing standardized measures to address management of non-tPA stroke (9).

We conclude that the impact of achieving primary stroke center certification is significant and has the potential to improve acute stroke care processes. We expect this to have beneficial effects on clinical outcomes based on prior clinical trials and observational studies.

## Data Availability Statement

The main FAST-MAG trial database and materials have been made publicly available at the NIH-NINDS Archived Clinical Research Datasets and can be accessed at https://www.ninds.nih.gov/Current-Research/Research-Funded-NINDS/Clinical-Research/Archived-Clinical-Research-Datasets. Supplementary data on clinically-recorded, ED-arrival GCS in FAST-MAG are available from author JS (jsaver@mednet.ucla.edu) upon reasonable request.

## Author Contributions

KS performed data acquisition, statistical analysis, drafting, writing, and revision of manuscript. TW and LV assisted with manuscript writing and data analysis. SH contributed to study design, statistical analysis, and revision of manuscript. SSta, ME, SStr, FP, and DL contributed to study design, data acquisition, and revision of manuscript. MK-T contributed to manuscript content planning, data analysis, and manuscript revision. RC contributions included funding and oversight. JS and NS contributed to study design, data acquisition, statistical analysis, writing, and revision of the manuscript.

### Conflict of Interest

The authors declare that the research was conducted in the absence of any commercial or financial relationships that could be construed as a potential conflict of interest.
